# Supporting the Community to Embrace Individuals with Dementia and to Be More Inclusive: Findings of a Conceptual Framework Development Study

**DOI:** 10.3390/ijerph191610335

**Published:** 2022-08-19

**Authors:** Valentina Bressan, Allette Snijder, Henriette Hansen, Kim Koldby, Knud Damgaard Andersen, Natalia Allegretti, Federica Porcu, Sara Marsillas, Alvaro García, Alvisa Palese

**Affiliations:** 1 Department of Medicine, University of Udine, Viale Ungheria 20, 33100 Udine, Italy; 2 Healthy Ageing Network Northern Netherlands, Peizerweg 140H, 9727 AP Groningen, The Netherlands; 3 South Denmark European Office, Av. Palmerston 18, 1000 Bruxelles, Belgium; 4 Department for Further Education, University College Lillebaelt, Niels Bohrs Allé 1, 5230 Odense, Denmark; 5 Odense Kommune, Department for the Elderly and Persons with Disabilities (ÆHF), Flakhaven 2, 5000 Odense, Denmark; 6 Connected Health Alliance CIC, 13A Ballyhoy Avenue, D05 K068 Dublin, Ireland; 7 Matia Gerontological Institute, Pinu Bidea, 35, 20018 Donostia-San Sebastián, Spain

**Keywords:** conceptual framework, community collaboration, dementia, family caregiver, people with dementia, policymaker

## Abstract

The number of community-dwelling people with dementia (PwD) is rising, and the role of their relatives is crucial in addressing and mitigating the implications of dementia on health care systems and on society. We developed a new conceptual framework to promote the collaboration of the community in supporting relatives who are caring for a PwD as well as a range of stakeholders in embracing dementia. A qualitatively driven, multi-method study divided into three phases was performed from 2019 to 2021. A qualitative descriptive study, a mixed-method systematic review and three consensus workshops were conducted, and their results were triangulated. The final version of the Community Collaboration Concept Framework is composed of three main domains based upon seven components: (1) embracing dementia; (2) creating empowerment and a sense of community; (3) collaborating through cocreation and design thinking. The new framework is based on the literature, the synthesis of empirical data and the consensus of a panel of international experts, supporting the global goal of improving community inclusiveness and collaboration. Further studies are needed to confirm its validity, how it should be implemented in practice in various settings and to propose improvements when designing projects based upon it.

## 1. Introduction

Worldwide, the incidence of age-related diseases, such as dementia, represents one of the primary health and social care concerns for society [1]. Approximately 47 million people are living with dementia (PwD), and it is estimated that there are nearly 10 million new cases every year [2]. Due to the rapid aging of the worldwide population, diagnoses of dementia are expected to triple over the next 30 years, with an increase in the socioeconomic burden on families, health and social care systems as well as for society as a whole [3].

In this scenario, the World Health Organization (WHO), with the support of Alzheimer’s Disease International (ADI), has considered dementia to be a public health priority, advocating for international and national actions based on inclusion, integration, equity and evidence-based principles [4]. Guided by this vision, many countries have initiated government programmes, plans and initiatives aimed at leading the social inclusion of PwD in their community, incorporating elements derived from dementia-friendly, dementia-capable and dementia-positive concepts [5]. Their main purpose is to guide the development of inclusive communities, where PwD and their family caregivers are supported, understood and engaged in society through a dynamic process of networks and meaningful relationships [6].

Different strategies are embodied in the abovementioned programmes such as promoting education and awareness regarding dementia across society to reduce the stigma and to improve social inclusion, acceptance and quality of life in PwD and their families [7]. Moreover, involving and maintaining engaged PwD in the community is a useful strategy to encourage them to remain living at home for as long as possible [7]. In this context, family carers are strategic in influencing positive outcomes for PwD (e.g., preventing hospital [8] and nursing homes admissions [9]) and to represent them in society when their direct involvement is no longer possible [10]. However, when family caregivers cannot manage their caregiving role, there is an increased risk of issues in health and social care systems, resulting in poor care at home, increased burden for the caregivers and frequent hospitalisation or avoidable institutionalisation. Therefore, the support offered to PwD by family caregivers should be considered as a national and international policy priority [11].

To date, different frameworks have been documented in the literature to promote the inclusion of PwD in society as well as strategies to provide services and resources through academic and community-based collaboration [12]. However, a specific conceptual framework to promote the collaboration of the community in supporting relatives in caring for a PwD is still lacking. In most Western countries, researchers and policymakers have explored and embraced the concept of “dementia-friendly communities” (DFCs), defined as places (e.g., towns, villages, organisations and groups) where PwD are empowered, understood, included and supported as an active part of the community [13]; however, its application is centred mainly on PwD and it is context dependent [14,15]. For example, while the WHO and the ADI consider DFC as an “approach” to normalise dementia in society [4], in the United Kingdom it has been applied as a policy initiative, a programme and a physical environment [5,15]. In the first case, WHO and ADI jointly presented a document describing a six-stage “acceptance of dementia” model to support the normalisation of dementia in communities around the world [5]. In the UK, the DFC approach has been incorporated in specific policies, achieving a formal recognition process with predetermined criteria and standards, while other countries have undertaken a less formal approach [15]. In addition, authors have underlined the risk that DFCs and dementia friends programmes may become paternalistic initiatives based on approaches focused on deficits caused by dementia rather than on strategies to enhance the resources of the person [16]. Therefore, despite the fact that the number of community-dwelling PwD is rising, the role of relatives of PwD remains crucial in addressing and mitigating the implications and consequences of dementia on health care systems and society [17]. Developing initiatives aimed at reinforcing awareness across different social sectors can support social acceptance and prevent stigma and the social exclusion of individuals with dementia [7]. Thus, continuing to develop conceptual frameworks to support the collaboration between family caregivers and communities might be useful for policymakers and providers. Promoting social inclusion, connection and participation for PwD and their caregivers also encourage their sense of belonging, community network and collaboration, safety, quality of life and well-being [7]. Furthermore, considering that stigma and social exclusion represent the main challenges for PwD living in the community and their relatives, community initiatives aimed at engaging and including both of them would seems strategic to help PwD to live in their domestic house for as long as possible. For these reasons, a multi-phase, qualitative study was conducted to generate a conceptual understanding of community collaboration based on the perspectives of relatives of PwD, health and social care professionals (HSCPs) and representatives of civil society organisations (CSOs), and to develop a related conceptual framework. Within a multi-phase research project (Embracing DEmeNtia (EDEN) http://embracingdementia.eu/, accessed on 18 July 2022 [18]), conducted in four European countries (i.e., Denmark, Italy, Spain and the Netherlands), the Community Collaboration Conceptual (CCC) Framework was developed and validated as a roadmap that provides guidance regarding how to design and implement activities and initiatives to support stakeholders, such as policymakers, volunteers, citizens, relatives, and HSCPs, to move from a desire to be a community embracing dementia to actually being this kind of community. This means that the CCC framework might represent a strategy for different stakeholders with a range of responsibilities at the local level (e.g., resources, expertise and ideas) to work together to promote more inclusive communities concerning PwD and their relatives by adopting a cooperative, creative and non-hierarchical approach.

## 2. Materials and Methods

**Figure 1:**
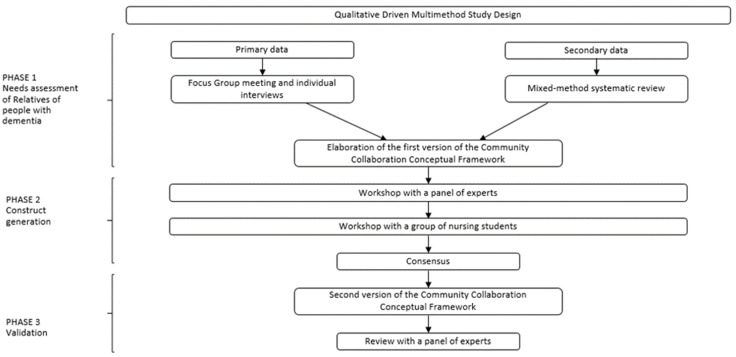
Process adopted to develop and validate the Community Collaboration Framework [21].

A qualitatively driven, multi-method design [19,20] divided into three phases (Figure 1) [21] consisting of multiple iterative phases of data collection, synthesis and validation was performed from January 2019 to December 2021. Three research methods, involving both a systematic review of the literature and qualitative research, were adopted. Specifically, our multi-method design was based upon: (a) a qualitative descriptive study; (b) a mixed-method systematic review; (c) a consensus conference method, which were performed rigorously and completed separately, and their results were triangulated [20] to develop the final framework.

### 2.1. Phase 1—Need Analysis

This phase was aimed at identifying (a) the needs and expectations of relatives of PwD and the most appropriate role and interventions expected by the community according to their experiences; (b) the strategies aimed at monitoring the processes and evaluating the outcomes of community interventions [22] in order to contribute to the CCC Framework’s development. During this phase, two different study designs were combined: (a) a qualitative study based on a deductive approach (top-down), collecting primary data; (b) a mixed-method systematic review based on an inductive (bottom-up) approach, collecting secondary data [23,24].

Specifically, between January and June 2019, a qualitative descriptive study was conducted to explore the needs, experiences, perspectives and expectations concerning the care of PwD at home [25]. We adopted a qualitative approach according to the evidence that the caregivers of PwD, when compared with caregivers of individuals with other kinds of health issues, show a higher risk of unmet needs, social isolation and low levels of quality of life, information and service access. Starting from these assumptions, primary data were collected from relatives of PwD, HSCPs and representatives of CSOs from four European countries (i.e., Denmark, Italy, Spain and the Netherlands) [25]. A purposive sampling strategy [26] was adopted to promote maximum variation in the caring experiences, socioeconomic and professional backgrounds, severity of the PwD’s disease and trajectory. We adopted a multiple recruitment strategy and potential participants were involved through the cooperation of Alzheimer’s/dementia societies, delegates of the CSOs and health care authorities at the national and/or regional levels of each country. Data were collected through 13 semistructured focus group (FG) discussions, integrated with 12 individual interviews conducted in parallel with the FGs by experienced and trained researchers in each country. The FGs and interviews were audio-recorded and then transcribed verbatim for the analysis. Some interviewees refused to be audio-recorded, and the researcher recorded information by collecting in-the-field notes. A total of 120 participants were involved: (a) 65 relatives of PwD (60 in the FGs, 5 in individual interviews); (b) 32 HSCPs (29 in FGs, 3 in individual interviews); (c) 23 members of CSOs (19 in FGs, 4 in individual interviews). Data collection was conducted in each country adopting a predefined interview guide exploring the needs, expectations and/or concerns that relatives taking care of a PwD experience on a daily basis while delivering informal care at home (more details are reported elsewhere [25]).

The data collected were analysed, abstracted and consolidated deductively [23] by the Netherlands partner, with the support of the research group.

Then, a mixed-method systematic review was conducted by searching and synthesising studies regarding the needs of relatives who take care of PwD at home [27]. The main aim of the review was to identify and synthetise the existing literature on the needs of family caregivers of people with dementia at home. This information was useful both for interpreting the results of the focus groups and interviews and for understanding PWD relatives’ needs and how they could be met by involving the community. The search terms included “caregivers”, “spouse”, “family members”, “informal caregivers”, “need”, “unmet need” and “dementia”, and they were applied in PubMed, the Cumulative Index to Nursing and Allied Health Literature, the Cochrane Database of Systematic Reviews and the PsycINFO database. Eligible studies were those with a quantitative, qualitative or mixed-method research design, published in English from 2009 to January 2019. A total of 1376 citations were initially identified through the search of the databases. After removing 180 titles and abstracts, 105 studies were selected. The full-text of these studies were read, and at the end of the selection process, 34 studies were included (more details are published elsewhere [27]). A data-based convergent synthesis approach was used to summarise the findings [28].

The results from the qualitative and the mixed-method systematic review informed the CCC framework domain development through a triangulation process to obtain corroborating evidence as it emerged in different research and data collection methods.

A first draft document that provided an overview of how the framework was developed, and a detailed description of its domain was presented and shared among the researchers in two online meetings. The first was conducted in November 2020, where the partners discussed and reviewed the emerging conceptual framework together. In the second, in January 2021, a first graphical representation of the CCC Framework was developed, adapting the Double Diamond Model created by the British Design Council [29] and embodying the consensus reached among researchers. This model is characterised by a flexible and straightforward nature; therefore, it is suitable for the context of dementia caregiving [30].

### 2.2. Phase 2—Construct Generation

In this phase, two key consultations with expert members and a group of future generations of HSCPs were performed, implementing a consensus method [31]. A consensus workshop was held in March 2021 with a group of 18 experts on care in dementia with different backgrounds (e.g., social workers, physicians and representatives of CSOs) and countries (e.g., Belgium, Spain and Luxemburg) according to James and Warren-Forward [32]. Participants were recruited considering their professional experience in the field of dementia and included academics, researchers, clinicians and policymakers from the CSOs and health care authorities at the national and/or regional levels of each country. The consensus conference technique was adopted to provide a deliberative participatory model designed to promote the development of a fact-finding procedure, where a set of best practices was discussed. The final intent was to ensure that researchers, policymakers and other stakeholders were supported to define balanced, credible, publicly legitimate and shared public health decisions [33,34,35].

Participants received the draft document describing the CCC Framework two weeks before the workshop. On the day of the meeting, after a brief explanation of the framework, participants were divided into five small groups and invited to collaboratively discuss, analyse and further develop this draft document. Each group was led by two researchers, and in order to promote the discussion, three main questions were asked, and the researchers recorded all group discussion by collecting in-the-field notes:

(a) Which factors can help to make the CCC Framework more process oriented?
(b) What kinds of strategies can be adopted to evaluate community collaboration activities?
(c) What factors/elements or strategies are needed to ensure the sustainability of the CCC Framework?

A second workshop was held in June 2021 with a group of 111 bachelor nursing students from second- and third-year courses. The intent was to validate the framework with the future generation of health care professionals. Students were prepared regarding the topic of dementia through theoretical and practical sessions, including lessons and clinical practice experience, laboratories and simulation scenarios, aimed at developing knowledge and competencies on PwD. In the current education pathway, they are not often engaged in cooperative activities that have been reported to increase knowledge and attitudes towards PwD [36]. For these reasons, we decided to involve nursing students, both as participants in the validation process of the framework and as learners to promote their substantial knowledge regarding the importance of community collaboration strategies, to improve the quality of care of PwD and their relatives.

The organisation feature of the meeting was the same as that organised for the experts: (a) students received a description of the CCC Framework two weeks before the meeting; (b) during the meeting, they were divided into 10 groups; (c) three main questions were posed during the groups’ discussion concerning (1) how to improve the framework, (2) which strategies will render the framework applicable, and (3) what strategies are needed to evaluate community collaboration activities. In both meetings, participants’ suggestions and recommendations were discussed in plenary sessions, and the CCC Framework draft was adjusted, obtaining a second version including the domains and components.

### 2.3. Phase 3—Validation

To validate the second version of the CCC Framework, a new consensus workshop was organised in October 2021 involving a panel of 16 experts from different backgrounds (e.g., social workers, physicians and representatives of CSOs) and countries according to James and Warren-Forward [32]. The experts received a detailed description of the last version of the CCC Framework two weeks before the workshop and, during the meeting, they were divided into five small discussion groups. Given that four experts were already involved in the previous phase, they were assigned to different groups. The experts were invited to analyse the domains and the components of the CCC Framework according to the following criteria: (a) their comprehensiveness; (b) their conceptual and descriptive clarity; (c) the level of abstraction and practical utility in the community, social and health care contexts. Qualitative notes were taken by the researchers during the meetings to refine the final version of the CCC Framework.

### 2.4. Ethical Considerations

According to the qualitative design adopted for the study and each country’s regulations, approval by ethics committee was not sought; however, all participants voluntarily gave their verbal and written consent before the FGs and individual interviews. Participants were assured that they could withdraw from the FGs and interviews at any time and that all data were collected and treated in order to guarantee confidentiality and anonymity, also regarding the quotes extracted. The consensus meetings were also conducted after having obtained the consent to participate from all members.

### 2.5. Rigour

During the phases of the CCC Framework’s development, all decisions and changes made were tracked, multiple data sources were used and continuing collaboration with participants was adopted [37]. In Phase 1, the quality of data collected was ensured by researchers using a guide addressing data collection processes and analysis procedures across countries. A specific categorisation matrix was also created to support the data analysis process, and all findings emerging from the national and cross-national analysis were discussed and counterchecked by all partners during a face-to-face meeting held at Udine University (IT) in June 2019 and in subsequent online meetings.

Regarding the mixed-method systematic review, the methodological quality of included studies was evaluated to explore their possible contribution to the synthesis [27]. Finally, in this phase, data triangulation was also adopted to promote representative credibility [31]. In Phases 2 and 3, a panel of experts from different backgrounds was involved, promoting reliability and reducing the risk of researcher bias.

## 3. Results

### 3.1. Phase 1—CCC Framework Core Elements

The qualitative study and mixed-method review revealed that, overall, relatives needed to receive accessible and tailored information, to be trained and educated to cope with changes in PwD and to find a balance between the needs of the PwD and their own personal needs. They also needed to be recognised for their caregiving role and be supported by specialised HSCPs [25,27]. Professionals who work in partnership with PwD and their relatives required a reorganisation of the available services, while CSOs were recognised as occupying a strategic role in compensating for the lack of support from formal services, encouraging networks, and promoting cooperation among stakeholders of local communities [25].

These findings supported the identification of seven core elements that guided the definition of the CCC Framework domains: (1) trusting in the health and social care system is crucial for relatives; (2) recognising, understanding and respecting the life conditions of relatives and PwD is a key point; (3) maintaining relationships with family members throughout the disease course is essential for HSCPs to ensure the quality and effectiveness of care; (4) valuing the role and the experience of CSOs is also strategic to ensure quality; (5) translating the available knowledge on dementia and how to cope with it into real-life situations, challenges, experiences and needs of PwD and relatives who live at home is necessary to develop effective support; (6) continuing to adapt knowledge to changes in needs and issues according to the different types of dementia; (7) helping people to better understand how stakeholders can participate in and support community collaboration by using narratives, videos and roleplaying as tools. The core elements were identified because they were evident and common in each of the FG discussions, individual interviews and in the literature review findings. The domains started to be identified by clustering the content of collected data into key concepts from core, nonoverlapping categories and as a result of the input of the experts’ discussion in the first workshop.

### 3.2. Phase 2—CCC Framework Generation

During Phase 2, the components and structure of the CCC Framework were further improved and enhanced and the main domains further defined.

### 3.3. Phase 3—CCC Framework Validation

**Figure 2:**
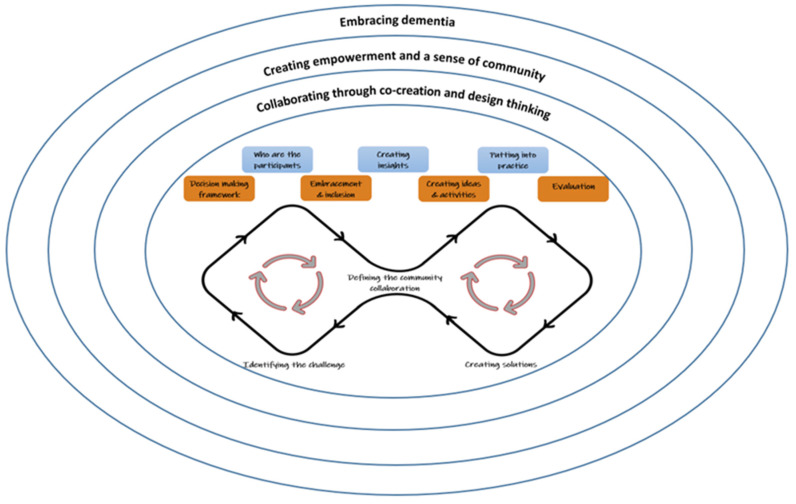
The Community Collaboration Conceptual Framework.

In this phase, the experts agreed that the contents of the framework were well described and structured and that their reciprocal connections were adequately defined. The domains and components showed logical congruence, while the low level of abstraction was a positive aspect, as the CCC Framework is characterised by concrete concepts. Overall, the experts judged the framework to be complete, clear and usable in community practice by professionals, relatives and political stakeholders. At the completion of this phase, the final version of the CCC Framework was defined and three main domains were identified:

(1) Embracing dementia.
(2) Creating empowerment and a sense of community.
(3) Collaborating through cocreation and design thinking (Figure 2).

Seven components were also identified as follows: (1) Decision-making framework; (2) Who are the participants; (3) Embracement and inclusion; (4) Creating insights; (5) Creating ideas and activities; (6) Putting into practice; (7) Evaluating.

#### 3.3.1. Embracing Dementia

A community embracing dementia refers both to a community of people brought together by geographical boundaries and a community of action aimed at building positive and supportive relationships regarding individuals with dementia and their relatives. This kind of community should be built on the close collaboration and transparent dialogue across stakeholders, with the support of politicians and/or local decision makers in order to develop concrete policies, strategies and interventions as well as to access financial and human resources.

#### 3.3.2. Creating Empowerment and a Sense of Community

When a community is empowered, people are encouraged to be engaged and active; they feel free to participate in society and, at the same time, feel a sense of belonging to it [38]. The meaningful involvement of PwD and their relatives into the development of community activities, initiatives and strategies is recommended to ensure these approaches are applicable, valuable and usable [7]. Therefore, to support the involvement of PwD in decision-making processes and improve their relationships and interaction, it is crucial to empower the community [39]. Through community participation and capacity-building, PwD and their relatives rediscover their own potential, gain confidence and broaden their (support) networks [40]. When seeking the empowerment of citizens, it is relevant to involve community members and groups, ensuring that they have real decision-making power and sufficient representation in the different community groups [41]. Every individual can collaborate according to his/her abilities and personal resources and participate with his/her own role and responsibilities. In this manner, all collaborating parties may feel heard and recognised for the knowledge, skills and experience they bring through their different roles [42].

#### 3.3.3. Collaborating through Cocreation and Design Thinking

Collaboration is a broadly utilised strategy for addressing complex social issues, such as dementia, and for promoting organisational innovation [43]; it focuses on identifying a common purpose and working towards joint decisions and being effective. Therefore, the process must be democratic and inclusive. The concept of collaboration is connected to the term “partnership”, which comprises alliances, groupings, associations and related forms of interorganisational relations aimed at improving health and well-being [44] for PwD and their relatives. According to Woodland and Hutton [43], the key components of collaborative initiatives are as follows: (1) having a shared purpose; (2) taking place in a nested way; (3) growing and developing in a predictable cycle; (4) being integrated and formalised on a continual system according to the complexity of the problem from somewhat integrated and informal to very combined and formalised; (5) being developed by collaborative teams.

When PwD, relatives and relevant stakeholders are brought together in constructive ways, adequately informed and included in a relevant context, they are able to create powerful visions and robust strategies for change. Therefore, the cocreation approach can be used to empower them to generate ideas and collaboratively create concepts, identify needs/aims, share knowledge and scientific evidence and balance this with what is possible in practice [45]. Cocreation is based on the belief that the users’ presence and contributions are essential in the creative process, as they provide insight into what is valuable to them adopting a cooperative and constructive relationship [46]. The end goal of cocreation is the same as that of research and concept design: to identify a solution that provides stakeholders with better experiences, and organisations with improved and innovative services [45].

Another solution can be the use of design thinking (DT), a problem-solving methodology that helps PwD relatives and relevant stakeholders to achieve a desired outcome related to a problem or to progress on a plan [47]. In health care, DT can be used as a method and practice of human-centred design to address complex challenges, capable of finding new solutions and expanding possibilities for action. [48]. It directs creativity towards goals, actions and (re)designing products, processes or services in an efficient and creative way, adopting a person-centred approach and considering real-world issues [49].

#### 3.3.4. The Components of the Community Collaboration Conceptual Framework

To serve the objective of the CCC Framework, seven components were identified (Table S1). The first four components (i.e., decision-making framework; who are the participants; embracement and inclusion; creating insights) belong to the first part of the double diamond model and are devoted to exploring the context, environment, people and knowledge available. They included strategies and activities devoted to identifying the challenges and potential obstacles to project development. The three last components (i.e., creating ideas and activities; putting into practice; evaluations) belong to the second part of the double diamond model and are devoted to finding and enacting creative solutions. The two circles inside the diamonds illustrate that (a) cocreation and design approaches are seldom linear processes; (b) different stakeholders can participate in the process at different stages, but all can contribute effectively also because of the avoidance of hierarchies; and (c) the model accepts a “fuzzy front end”, and the stakeholders are allowed to imagine, create and test solutions and activities that do not yet exist.

Within the proposed CCC Framework, the components represent a sort of map that users can adopt to organise their thoughts to improve the creative process, adopting a nonlinear approach where there is no predefined starting and ending points, but the process can be undertaken from any point. They are encouraged to go back and forth between components in order to fully understand the potential of the community and resources already available as well as to design projects and strategies to develop collaboration within the community. Adopting these methodologies, users can also understand any problems and obstacles and how they can either solve them or improve upon an existing solution.

## 4. Discussion

### 4.1. Methodological Discussion

The research process involved several countries, partners and experts in multiple meetings and steps. The qualitative study regarding the needs, experiences and expectations [25] was performed before the mixed-method systematic review, thus focusing on the lived experiences first and then considering the evidence already available. This has increased the degree of familiarity of partners regarding the topic under consideration and has also promoted immediate engagement with the stakeholders further involved in the entire project. Moreover, at the overall level, a multi-method design was used, combining focus group discussions, individual interviews, panel discussions and a systematic review of the literature, increasing the reliability of the information and benefitting from a wider perspective. However, the framework needs to be implemented in practice to be refined and redefined.

### 4.2. Findings Discussion

We developed a conceptual framework that can guide and support those communities that want to be more inclusive with regards to PwD and their relatives. It incorporates evidence from the literature and the direct experiences of relevant stakeholders regarding how society can be inclusive; in addition, it includes strategies and actions on how people can collaborate to improve the quality of life of PwD and their relatives as part of the conceptual representation. We started by analysing the ideal concept of community collaboration [50] and that of DFCs [4] to develop a new conceptualisation. The CCC Framework incorporates the principles of these two concepts to promote the involvement of different organisations and the roles of individual representatives [50] by emphasising the centrality of PwD [51]. However, it is based on the analysis of the needs of relatives of PwD and a multi-stakeholder partnership through a collaborative and co-creative process.

The CCC Framework aims to offer the opportunity to develop projects and initiatives adopting a holistic and comprehensive view: stakeholders are directly invited to design projects and to evaluate their outcomes including the identification of effective interventions, understanding the process of implementation and, finally, evaluating the interventions’ sustainability. In this context, the CCC Framework could be used by HSCPs and policymakers to guide decisions regarding aspects of services that need to be developed or redesigned. Moreover, it could be useful to promote inclusive beliefs and values across the community and to create a shared vision for a more collaborative community (e.g., how to encourage relatives and PwD to participate in social activities, using and promoting their capabilities and empowering their potentialities).

The CCC Framework also tries to overcome the sometimes-paternalistic approach of the dementia-friendly community concept [16] and to promote a more family-centred approach: it puts the relatives and their needs at the centre of the whole process of project development, considering them to be experienced in the challenges and key elements of a sustainable care system.

According to the framework, relatives and stakeholders are invited to participate and co-collaborate following an ongoing process characterised by being dynamic, adhering to the changing needs of relatives of PwD, responding with community resources and supported by a bottom-up approach [52]. The intent is to promote confidence and competence in participants, enhance the development of self-awareness regarding the issues related to dementia and to empower the community. The adoption of the cocreation and codesign approaches also allows for PwD, relatives and relevant stakeholders to be fully involved in the projects’ development, be engaged in a shared decision-making process and ensures the outcomes meet the needs of the target population [53]. In this context, the cocreation approach allows for the sustainability of a project thanks to the combination of available resources, knowledge and capabilities of multiple stakeholders and the promotion of social responsibility [54]. The CCC Framework can also be considered a strategy to better support the development of a community that wants to move from a desire to be a community embracing dementia to being this kind of community.

Another important characteristic of the new Framework regards the evaluation strategies. These are derived from the needs analysis process and are based on four main challenges that are the most important for relatives of PwD to manage and to receive support. These challenges are also indicators of a community’s ability to collaborate and be inclusive, to understand and to cope with the challenges imposed by the dementia. The evaluation strategies should be adopted as a pragmatic approach to identifying a set of comprehensive indicators to monitor the capacity of multiple stakeholders to work in partnership and to achieve the expected outcomes. These strategies provide guidance for comprehensive community or context evaluation through which the users can identify, adapt and test specific indicators in practice. Following the suggestions of Heath and Frey [50], the new framework has been designed considering that the choice of a community to become inclusive towards PwD and their relatives depends on the maturity of its members. The maturity of an inclusive community can be observed in the degree to which the PwD and their relatives can access services and social events, interact with other members and be involved in valued and targeted activities [55], specifically, some rural and small communities where every individual knows each other and provide support and access to services when members notice that they are needed by someone [56]. In larger communities, many people can recognise whether a PwD needs help if they are involved in awareness-raising activities [57]. In more mature communities, activities and programmes focus on inclusiveness and are supported by well-established policies, economic investment, social initiatives and an integration of social community activities with the health care system [55].

Where awareness of dementia and social inclusion needs to be raised, the CCC Framework can be used to define an action plan to identify efforts that can be adopted to develop projects aimed at transforming the community, identifying founding sources, promoting project sustainability, and identifying the main stakeholders. In communities where social inclusion of PwD is already present, the CCC Framework can stimulate stakeholders’ discussion on how, for example, to integrate and improve dementia-friendly, age-friendly and community collaboration initiatives already in use and to create new ones.

Furthermore, thinking about a wider use of the Framework, it can also be used as a tool by HSCPs to move beyond a focus on a list of professionals’ tasks and technical skills, offering theoretical and practical opportunities to improve the customised care of PwD and the partnership with their relatives in the care plan. In this case, the framework could also support the measurement of the success of this involvement in terms of outcomes, creating a sort of standardisation [58]. For policy - and decisionmakers, the Framework could be considered as a starting point for designing interventions and developing policies and strategies aimed at improving the empowerment, engagement and active participation in community activities of PwD and their relatives. This point is crucial because sustainability and financial support often represent a critical issue and cause concern amongst stakeholders. Local authorities and policymakers commonly question why and how they should invest in a project, and what are the expected challenges and outcomes. The CCC Framework could help to answer these questions, engage key stakeholders and generate good, shared examples through documenting a complete pathway of implementation. It also could offer strategies to support stakeholders to self-assess the strengths and limitations of their projects, to support programme implementation and to check the parts of the former process and evaluate their effectiveness.

### 4.3. Limitations

The methodology adopted has several limitations.

Regarding the needs analysis (Phase 1), multiple strategy sampling recruitment was adopted to ensure participant variation; however, social and cultural differences both at the societal, community and health care system levels across the countries may have influenced both the process and the findings.

For the literature review, only primary studies published in indexed journals and written in English were selected, while the grey literature was excluded, introducing a potential information bias. In Phase 2, a large group of participants were involved in the consensus meetings despite the optimal panel size having been established as being between 6 and 12 participants [31]. To ensure that all participants could contribute to the discussion and to manage any potential conflicts properly, participants were divided into small groups of discussion facilitated by an experienced researcher [30]. Finally, in the validation of the second version of the CCC Framework (Phase 3), only four experts attended the previous phase of the process according to their willingness to participate. The process undertaken was time consuming and, therefore, experts declined to participate. On the one hand, this increased the number of experts involved, thus promoting ample participation in the process; on the other hand, this might have influenced the findings.

## 5. Conclusions

In this study, we presented and discussed the process undertaken to develop and validated a conceptual Framework aimed at improving community collaboration and inclusion with PwD and their relatives. The Framework was based on the evidence available, as reported in the literature, and on the synthesis of empirical data and consensus of experts. Its intent is to support the global goal of using a family-centred approach and improving community inclusiveness and collaboration. The implementation of the CCC Framework will, in the future, lead to changes in organisational policies and support further research at the national and international levels. However, to confirm the validity of the Framework and how it should be used in practice, further research should be conducted on its use in various settings, and improvements should be proposed when designing projects based on it, implementing interventions and evaluating the outcomes in the specific context where they are applied. These local experiences might add further insights to better conceptualise community collaboration in the field of dementia care and generate practical tools and initiatives to support its implementation. Research will also be needed to review and update the framework over time, and stakeholders may be able to use it to plan an implementation roadmap and as a tool for negotiating with funders and other key stakeholders. The development of benchmarking initiatives for the application of the new framework across European countries and regions promoting specific research projects and shared initiatives are also suggested.

## Data Availability

The data presented in this study are available on request from the corresponding author.

## Supplementary Materials

The following supporting information can be downloaded at: https://www.mdpi.com/article/10.3390/ijerph191610335/s1, Table S1: Description of the components of the Community Collaboration Conceptual Framework.
