# Supplementary material for: Supporting the Community to Embrace Individuals with Dementia and to Be More Inclusive: Findings of a Conceptual Framework Development Study
Source: Int J Environ Res Public Health. 2022 Aug 19;19(16):10335. doi: 10.3390/ijerph191610335 (PMC9407991; doi:10.3390/ijerph191610335)
Supplement: Supplementary file 1 [file ijerph-19-10335-s001.zip › ijerph-1849063-supplementary.pdf]

Table S1. Description of the components of the Community Collaboration Conceptual Framework

Components	Descriptions
(1) Decision-making framework	It is strategic to motivate and support the creation of an overall vision for active ageing and promotion of a community embracing dementia in policy. Politicians and local decision makers are crucial in promoting inclusive and supportive communities for PwD and their relatives. It is also important to engage with agencies and other stakeholders to support projects and programmes as well as to promote local and national-level policies that support community collaboration and inclusiveness.
(2) Who are the participants	The model can be used by family members, HSCPs, CSOs, volunteers and individual citizens who want to propose and implement initiatives and projects in the context of dementia and social inclusiveness. It is important to create an overview of the relevant stakeholders and designate roles to the different participants in the process. This allows the creation of collaborative groups where members are both representative of the group and actively work on the projects (Heath & Frey, 2004).
(3) Embracement & inclusion	There is a need to understand what makes a community that embraces dementia, and promote strategies aimed at insuring that PwD and their relatives can stay included in the community and continue living an active life for as long as possible.
(4) Creating insights	It is necessary to know about the group of people who should benefit from the changes, and ask open questions that can provide the right insight into the process. The goals are: 1) to improve society collaboration and help focus attention on what is important in the life of PwD and their relatives; and 2) identify what can be done to guarantee that PwD and their relatives' have greater control and a higher level of happiness and well-being.
(5) Creating ideas & activities	When creating and developing ideas and activities, it is important to take into account the starting point from the insights that were gathered in the previous section. It is also important to consider the institutions and subjects that develop them, possibly by adopting approaches based on co-creation. Some key elements of these approaches are: 1) seek and enhance the experience of end users; 2) identify problems together; 3) use typical methods of participatory action research; and 4) focus attention on the development of solutions.
(6) Putting into practice	Defining a concise and effective implementation plan is important to bring clarity to the overall vision and determine the purpose and goals of the different activities to be implemented within the co-creation process. Action planning has several advantages, helping to develop ideas and thoughts and implement interventions.
(7) Evaluation	As part of the co-creation process, stakeholders should, in collaboration with PwD and their relatives, set up a number of concrete indicators. These need to be based on local and specific conditions, cultures, structures and projects. Evaluation is an important element of planning because it allows you to check whether the project is achieving the established objectives and helps to improve future initiatives based on what has been learned. In order to ensure a continuous evaluation system, the evaluation criteria should be defined before the implementation of the project and the development of result indicators can be used to foresee the evaluation of the following aspects: 1) being able to face the future; 2) support and collaboration with PwD, relatives and key stakeholders; 3) becoming or staying socially included; and 4) understanding and coping with dementia.

Note: CSOs, civil society organisations; HSCP, health and social care professionals; PwD, people with dementia
